# Interconnections between the Gut Microbiome and Alzheimer’s Disease: Mechanisms and Therapeutic Potential

**DOI:** 10.3390/ijms25168619

**Published:** 2024-08-07

**Authors:** Ahmad M. Sait, Philip J. R. Day

**Affiliations:** 1 Medical Laboratory Science, Faculty of Applied Medical Science, King Abdulaziz University, Jeddah 21589, Saudi Arabia; ammsait@kau.edu.sa; 2 Regenerative Medicine Unit, King Fahd Medical Research Center, King Abdulaziz University, Jeddah 21589, Saudi Arabia; 3 Division of Evolution and Genomic Sciences, Faculty of Biology, Medicine and Health, The University of Manchester, Manchester M13 9PL, UK; 4 Department of Medicine, University of Cape Town, Cape Town 7925, South Africa

**Keywords:** gut microbiome, Alzheimer’s disease (AD), lipopolysaccharide (LPS), short-chain fatty acids (SCFAs)

## Abstract

Alzheimer’s disease (AD) is a neurodegenerative disease that is known to accumulate amyloid-β (Aβ) and tau protein. Clinical studies have not identified pathogenesis mechanisms or produced an effective cure for AD. The Aβ monoclonal antibody lecanemab reduces Aβ plaque formation for the treatment of AD, but more studies are required to increase the effectiveness of drugs to reduce cognitive decline. The lack of AD therapy targets and evidence of an association with an acute neuroinflammatory response caused by several bacteria and viruses in some individuals has led to the establishment of the infection hypothesis during the last 10 years. How pathogens cross the blood–brain barrier is highly topical and is seen to be pivotal in proving the hypothesis. This review summarizes the possible role of the gut microbiome in the pathogenesis of AD and feasible therapeutic approaches and current research limitations.

## 1. Introduction

AD is the predominant neurodegenerative illness, affecting millions of people around the globe [1]. Its impact on memory, cognition, and quality of life makes understanding the intricacies of the disease not just a scientific pursuit, but it also has a humanitarian basis [2]. AD significantly impacts caregivers, leading to heightened burden and depression due to the behavioural and psychological symptoms displayed by those with the condition [3]. Based on the Medicare Current Beneficiary Survey and research on disease prevalence, it is expected that the total direct costs for AD care will increase from USD 226 billion in 2015 to USD 1.1 trillion by 2050 [4]. The main pathological hallmarks for AD are the accumulation of Aβ and hyperphosphorylation of tau protein [5]. AD primarily has two main types: (1) sporadic and recognized as late-onset AD (LOAD) and (2) early-onset AD (EOAD) [6,7]. There is no cure yet for AD; however, treatments used to alleviate the symptoms include donepezil, rivastigmine, and memantine [8]. The causative agent of AD remains unknown; therefore, there is an urgent need to explore innovative pathways to investigate its complex pathogenicity [1]. Central to this understanding is the developing research on the gut microbiome, which plays a pivotal role in overall health and disease. Recent studies have begun to unravel a fascinating connection between the gut microbiome and AD [9,10,11]. This relationship is coordinated through the brain–gut axis, a complex network that allows for bidirectional communication between the central nervous system and the gastrointestinal tract [12]. This axis suggests a pathway through which the gut microbiome influences the development of neurodegenerative diseases, including AD [13]. Numerous research databases were used in this review, including PubMed, Web of Science, and Scopus. This paper discusses the sophisticated relationship between the gut microbiome and AD. It explores the diverse gut microbiome communities detected in AD patients, the influence of short-chain fatty acids and neurotransmitters, and the potential role of lipopolysaccharides (LPSs) in amyloid accumulation. Also, it explores the notion of a ‘leaky gut’ and how subsequent inflammation might contribute to the pathophysiology of AD. Additionally, possible treatment methods that include using prebiotics, synbiotics, and antibiotics are discussed. Understanding these mechanisms opens the door to novel treatment possibilities, leveraging the gut microbiome to combat AD.

## 2. How the Gut and the Brain Communicate

The gut–brain axis (GBA) can be defined as bidirectional communication that links the gut with the brain, connecting the emotion and cognition in the brain with the gut [14]. This form of communication can be facilitated via the vagus nerve, brain, spinal cord, enteric nervous system (ENS), hypothalamic–pituitary–adrenal (HPA) axis, and autonomic nervous system (ANS) [15]. In addition, this bidirectional signalling occurs by neurotransmitters, short fatty acid chains, cytokines, and microbial metabolites [16]. The disturbance of this communication has been involved in many types of neurological diseases including Alzheimer’s and Parkinson’s [17].

## 3. A Possible Gut Microbiome Link to AD

**Figure 1:**
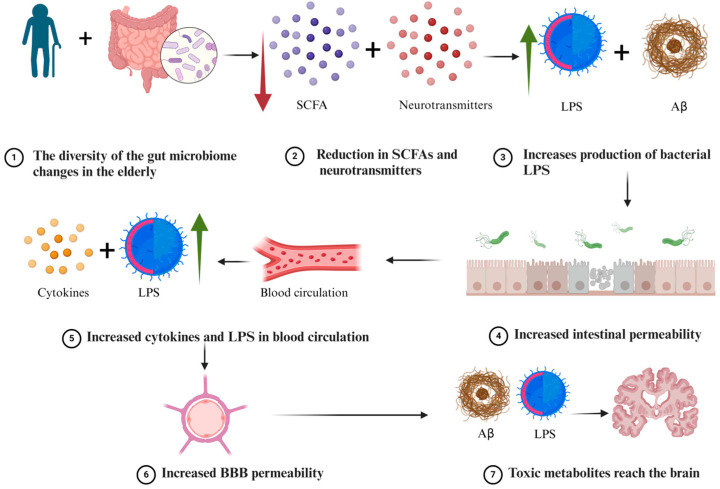
A possible pathway of how the gut microbiome contributes to AD in the elderly.

Ageing is one of the main risk factors related to AD [18]. Throughout life, the gut microbiome community has been proven to change as a function of age [19]. Therefore, these changes are not favourable for the elderly [20]. The alteration in the gut microbiome community reduces the levels of neurotransmitters, short-chain fatty acids (SCFAs), and increases the production of Aβ and LPSs [20]. These microbial excretions can penetrate the epithelium to reach the blood circulation and cross the blood–brain barrier (BBB). Hence, the integrity of the BBB is impaired due to ageing and the reduced secretion of SCFAs that is required for the BBB to function [21]. This scenario will lead to systemic inflammation that enhances the permeability of the BBB, resulting in increased LPSs and Aβ to reach the brain and promote the progression of AD [22] (Figure 1).

## 4. The Gut Microbiome Community in AD Patients

Numerous studies investigated the microbiome community in AD compared to controls and found alterations in the AD gut microbiome (Table 1). For instance, Zhuang et al. (2018) analysed faecal samples from 43 AD patients and 43 age-matched control subjects [23]. The findings revealed that the AD patients exhibited a higher presence of *Bacteroidetes* and a reduced level of *Actinobacteria* in comparison to the control group. These differences in both phylum groups were statistically significant. Liu et al. (2019) examined the gut microbiome in 33 AD patients and 32 control individuals. The results indicated a decrease in *Firmicutes*, a vital phylum known for producing SCFAs, in the AD patients relative to the control group [24]. Furthermore, the level of *Proteobacteria* was found to be higher in AD patients compared to the control group. The previous two studies were performed in a Chinese population and yet in AD patients identified varying dominant bacterial strains. However, the small sample size in each study could be a factor affecting the results. To gain a clearer insight into the distribution of gut bacterial strains in AD patients, studies with larger participant numbers are necessary.

Verhaar et al. (2022) conducted a study on the gut microbiome across three patient groups, including 33 with AD, 21 with Mild Cognitive Impairment (MCI), and 116 with Subjective Cognitive Decline (SCD) [13]. The study involved comparing the top 20 bacterial genera found in these groups. The results indicated that only two genera, *Subdoligranulum* and *Phascolarctobacterium*, both belonging to the phylum *Firmicutes*, showed statistically significant differences. Vogt et al. (2017) investigated the diversity of the gut microbiome in a group of 25 AD patients and compared it with 25 healthy individuals [25]. The results indicated that in the AD patients, there was a decrease in *Firmicutes* and *Bifidobacterium* and an increase in *Bacteroidetes*, when compared to the healthy controls. These differences were statistically significant.

Considering the cumulative data from various studies conducted globally, including in China, the Netherlands, and the United States of America, a distinct variation in the microbiome community is evident [13,23,24]. Even within identical communities, the diversity of the microbiome can vary significantly. Numerous factors influence this diversity such as age, delivery mode, and genetics [26,27,28,29], highlighting the necessity for more extensive and comprehensive studies with larger participant groups to accurately represent the state of this microbial diversity.

## 5. Short-Chain Fatty Acids

SCFAs, synthesized by gut bacteria from dietary fibres, are composed of a minimum of six carbon atoms [29]. Numerous varieties of SCFAs exist, such as formate, acetate, propionate, butyrate, valerate, and caproate [30]. It was demonstrated that SCFAs have many essential functions such as the ability to induce reactive oxygen species (ROS), alter the proliferation rate, have antimicrobial effects, and lastly, it can change the gut microbiome community [31]. The absorption of SCFAs can be through the mucous epithelium of the caecum and colon [30]. However, SCFAs have the ability to directly enter the blood circulation and cross the BBB [32] through lipid bilayer permeability.

**Figure 2:**
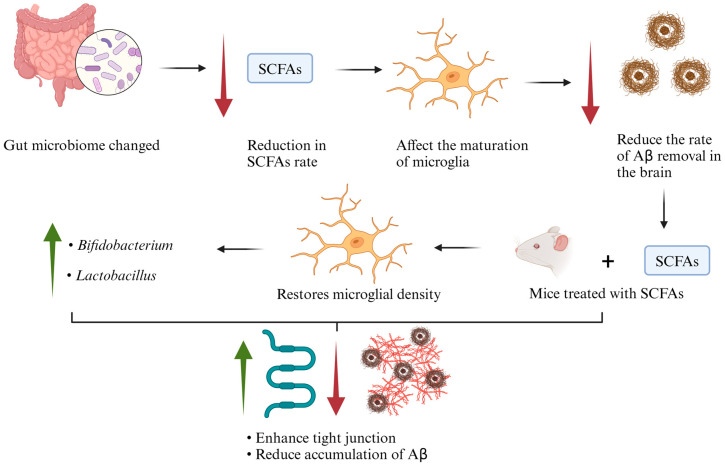
SCFAs in mice enhanced the tight junction and reduced the accumulation of Aβ.

In AD, microglial cells are a key element involved in the removal of Aβ from the brain [33]. SCFAs are crucial in the maturation process of microglial cells. For instance, Erny et al. (2015) investigated the role of SCFAs in mice free from gut microbiota to understand if SCFAs could restore the function of microglia; they added SCFA mix in the drinking water of germ-free mice for 4 weeks [34]. The outcomes showed normalized microglial density and normal numbers of segments and branching points (Figure 2). Therefore, if the production of SCFAs is disrupted, it impacts the rate of Aβ clearance from the brain by influencing microglia, which play a crucial role in Aβ removal [35,36]. This disruption may lead to an increased accumulation of Aβ and subsequent progression of cognitive decline (Figure 2). Moreover, Zajac et al. (2022) investigated the effect of SCFAs in APPswe/PSEN1dE9 mice, where mice were treated with SCFAs (acetate, propionate, butyrate, and chloride) for five months [36]. They evaluated microbiome diversity, memory impairment, and astrocyte activation by the expression of glial fibrillar acidic protein (Gfap) and amyloid expression. The results indicated a significant increase in alpha diversity due to SCFAs in male mice at the order, class, and phylum levels. In female mice, a significant elevation was observed at the genus level. There was an increase in bacteria involved in the production of SCFAs, in male mice *Bifidobacterium*, and in female mice *Lactobacillus* (Figure 2). There was no effect on the expression of Gfap caused by SCFAs. In addition, SCFAs did not improve the cognitive function of the mice. Furthermore, there was no significant effect on the cortex or hippocampus for amyloid β 40 (Aβ40). The level of amyloid β 42 (Aβ42) was also not affected by the treatment of SCFAs on the cortex or hippocampus [36].

Colombo et al. (2021) found that SCFAs promote the aggregation of Aβ. They used three main types of mice, (1) germ-free amyloid precursor protein (APP) and presenilin-1 (PS1) genes (APPPS1) (control), (2) GF APPPS1, naturally recolonized, and (3) GF APPPS1, specific pathogen-free (SPF) [9]. They measured the Aβ accumulation levels, and the outcomes revealed that there was a reduction in Aβ in GF APPPS1 animals compared to other experimental groups (i.e., groups 2 and 3). Interestingly, treatment with SCFAs (propionate, butyrate, acetate) for group 3 led to an increase in the accumulation of Aβ, and the results were statistically significant compared to groups 1 and 2 [9].

Xie et al. (2023) examined the link between the gut microbiome and the integrity of blood cerebrospinal fluid (CSF) associated with tight junctions. They used four main mice groups, (1) germ-free (GF) mice, (2) recolonized GF (GFR), (3) broad-spectrum antibiotic-treated (AB), (4) recolonized AB mice (ABR), and (5) generated gut microbiota-depleted mice by giving SPF [37]. The immunofluorescence assay showed low expression and change in the localization for zona occludens-1 (ZO-1) (critical in maintaining the endothelial cells in the BBB) and occludin (OCLN) (regulates the stability of the tight junctions), in the brain vessels of groups 1 and 3 compared to group 5 [38,39,40]. The recolonized AB group restored the integrity of ZO-1 and OCLN. In addition, they administrated SCFAs (propionate and butyrate) to APP^NL-G-F^ mice (AD model that was shown to have reduced BBB permeability and a reduced rate of bacteria-generating SCFAs). The results indicated an enhancement in the subcellular localization of tight junctions at the blood–CSF barrier and a reduction in Aβ accumulation (Figure 2) [37].

## 6. Neurotransmitters

Neurotransmitters, chemicals transported across synapses between neurons, convey messages that regulate behaviours including motility, emotion, and memory [40]. The operational functions of the brain are dependent on the neurotransmission between different types of neurons and glial cells [41]. Disproportions in these neurotransmitters can result in neurological and psychological conditions, including AD, Parkinson’s disease, autism spectrum disorder, anxiety disorders, and depressive disorders [42].

**Figure 3:**
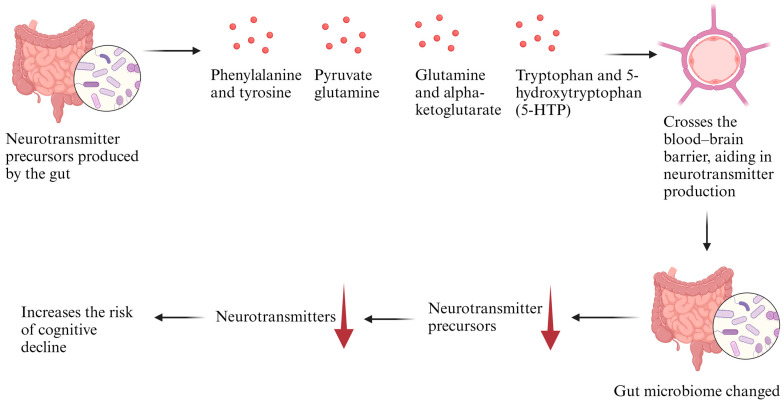
Gut microbiome’s role in neurotransmitter production and cognitive decline.

There are two main ways neurotransmitters can affect the biological metabolite of the brain: (1) When there is an alteration in the level of the neurotransmitters that affect the communication to the brain by the vagus nerve [42,43]. For example, the neurons in the nucleus tractus solitarius (NTS) can form synaptic connections with enteroendocrine cells and neurotransmitters and purinergic signalling that transmit to the brain and contribute to the regulation of microglia cells [44,45]. (2) Many researchers have associated the role of the gut microbiome to impact the production of serotonin, gamma-aminobutyric acid (GABA), glutamate, and dopamine [46,47,48]. Since neurotransmitters cannot cross the BBB, but their precursors can, under normal conditions, there is an accumulation of the precursors involved in the production of the neurotransmitters [40]. If the gut microbiome changes and alters the rate of the neurotransmitters produced, then that in turn affects the rate of precursors accumulated in the brain, leading to a reduced amount of neurotransmitter production and less neuronal communications [47] (Figure 3). The level of these neurotransmitters was altered and reduced in AD patients as revealed by many studies (serotonin [48]), (GABA [49]), (glutamate [50]), (dopamine [46]). In addition, Liu et al. (2021) identified a correlation between the modification of precursors used in the creation of neurotransmitters and the disruption in the synthesis and control of various vital neurotransmitters in patients with AD [51]. Other research indicated a direct relationship between neurotransmitters and the synthesis of neurons in the brain. For example, Liu et al. (2009) showed an increase in the development of enteric neurons two to three weeks after treatment with a serotonin 5-HT4 agonist [52]. Additionally, it was found that neurons in GF mice, which cannot synthesize serotonin, showed less development [52].

## 7. The Role of the Lipopolysaccharide

LPSs constitute a key element of the outer membrane of the cell wall in Gram-negative bacteria [53]. An earlier report shows that the level of blood LPSs was higher by 3-fold in AD patients compared to the control [54]. In addition, many bacterial strains that produce LPSs were found to be present in AD patients including *Bacteroides fragilis* and *Escherichia coli* [55,56]. Zhao et al. (2017) observed that in AD patients, LPSs accumulate in the neuronal parenchyma and are found around the edges of the neuronal nuclei [57]. In addition, LPSs have been shown to induce many pathological markers related to AD in animal models including the induction of amyloid and tau aggregation, tau phosphorylation, and reducing synaptic plasticity [58,59,60].

Marizzoni et al. (2023) investigated many biomarkers related to the gut microbiome in cognitive impairment patients due to AD, *n* = 34, and compared them to the control, *n* = 13 [60]. They found that LPSs were elevated in patients with AD compared to the control. In addition, their findings supported that LPSs translocate from the gut to the bloodstream as result of increased gut permeability. They measured many important proteins that are critical to the gut endothelium. For example, they found that platelet endothelial cell adhesion molecule-1 (PECAM-1) and platelet selectin (P-selectin) were significantly increased in AD patients compared to the controls. The upregulation of cell adhesion molecules contributes to the response against infection and controlling leukocyte distribution and vascular inflammation [60].

**Figure 4:**
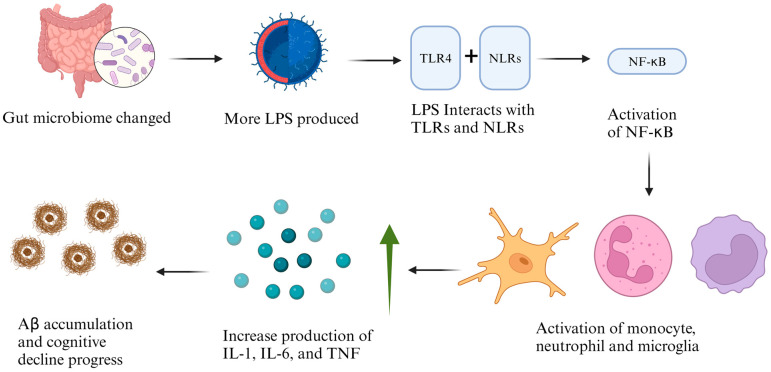
The role of LPSs in the gut microbiome and cognitive decline.

LPSs possess the capability to stimulate the immune system by interacting with Toll-like receptors (TLRs) and NOD-like receptors (NLRs), thereby activating nuclear factor kappa B (NF-κB) [61]. This activation in monocytes, neutrophils, and microglia enhances the production of key cytokines such as Interleukin-1 (IL-1), Interleukin-6 (IL-6), and tumour necrosis factor (TNF) [20]. These cytokines contribute to an increase in Aβ production through the regulation of β-amyloid precursor protein (β-APP) mRNA levels and the activity of β-secretase 1 (BACE1) [20] (Figure 4). Furthermore, LPSs can compromise the integrity of the BBB, leading to the reduced clearance of amyloid beta from the brain and consequently causing its accumulation [21]. Recently, Tejera et al. (2019) induced inflammation by the LPS in APP/PS1 mice and found a reduction in the clearance of amyloid beta by activating nod-like receptor pyrin 3 (NLRP3) [62]. Remarkably, it was reported that the LPS was found to be localized within Aβ using immunofluorescence [55].

## 8. Amyloid Production

Many bacterial species that reside in the gut under normal conditions can produce Aβ such as *Escherichia coli* and *Bacillus subtilis* [63]. Bacterial amyloids can have the same effects as central nervous system amyloids and can cross the BBB and reach the brain to contribute to AD pathogenesis. Harach et al. (2017) showed that amyloids produced by gut bacteria influence cerebral amyloids [64].

Sheng et al. (2022) investigated the gut microbiome species using 16S ribosomal RNA (rRNA) sequencing [65]. In addition, they evaluated the plasma level for amyloid betas Aβ40, Aβ42, and Aβ40/Aβ42. The study focused on both Aβ-positive and Aβ-negative cognitively normal individuals. The results indicated a lower plasma level of Aβ42 in patients positive for Aβ compared to those negative for Aβ. Moreover, there was a notable increase in *Bacteroidetes*, while both *Firmicutes* and *Deltaproteobacteria* showed significant reductions. An increase in *Bacteroidetes* potentially elevates LPS production, initiating inflammation and raising its levels in the bloodstream, which then may reach the brain and cause additional inflammation. Conversely, a decrease in *Firmicutes* leads to a reduced production of SCFAs, which are vital for maintaining the integrity of the BBB and the maturation of microglial cells [65].

Das et al. (2022) found that bacterial amyloids led to an increase in TLR2 levels in the gut of a transgenic AD model when compared to the wild type [66]. Furthermore, the response of the vagus nerve to bacterial amyloids was assessed using PGP9.5, showing colocalization with elevated TLR2 expression. Additional testing with a 3D human ileal mini-gut monolayer in vitro model also demonstrated an increase in TLR2 expression [66].

## 9. Leaky Gut and Inflammation

The intestinal mucosal barrier is a crucial defence mechanism that prevents toxins from entering the bloodstream [67]. However, factors like microbial infections, immune disorders, or conditions such as inflammatory bowel disease (IBD) can increase the permeability of this barrier [68]. This increased permeability allows more toxins to enter the bloodstream, potentially leading to widespread inflammation [69]. Research conducted by Stadlbauer et al. (2020) focused on the gut microbiome and its link to dementia [70]. The study found that gut bacteria dysbiosis is associated with dementia. This imbalance was characterized by changes in the diversity and composition of gut bacteria. It also resulted in increased gut permeability, as indicated by higher serum diamine oxidase (DAO) levels, and systemic inflammation, shown by elevated levels of soluble cluster of differentiation 14 (sCD14) [70]. Additionally, a decrease in the *Lachnospiraceae* NK4A136 group, known for butyrate production, was observed in individuals with dementia. External factors such as malnutrition and medication intake were noted to influence the gut microbiome [71,72,73].

**Figure 5:**
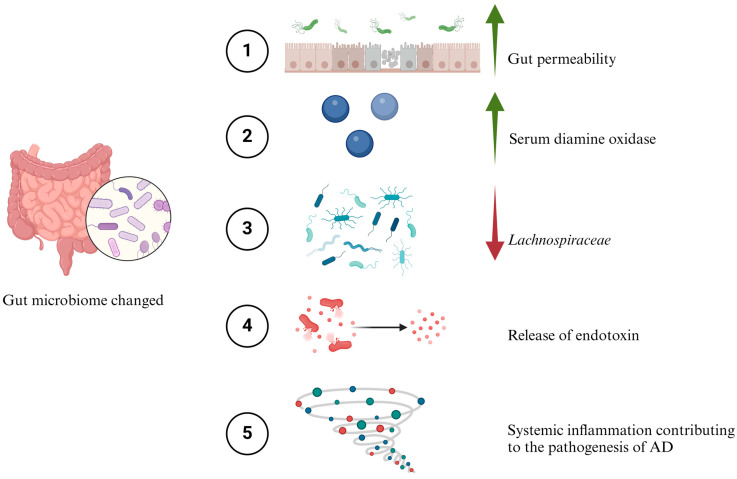
Role of inflammation and gut microbiome in pathogenesis of AD. (1) An imbalance in the gut microbiome, known as dysbiosis, leads to increased intestinal permeability. (2) There is an obvious rise in serum diamine oxidase levels, a biomarker indicating this increased permeability of the intestine. (3) A significant reduction is observed in the *Lachnospiraceae* bacterial strain, which is crucial for butyrate production. (4) Additionally, there is an elevation in endotoxin levels originating from the gut microbiome. (5) These changes collectively may contribute to systemic inflammation, a key risk factor in the development of AD.

The increase in intestinal permeability also raises the likelihood of systemic inflammation [73]. Endotoxins, for instance, can trigger such inflammation; they activate TLRs on cell membranes, which recognize inflammatory molecules known as “pattern-associated molecular patterns” (PAMPs). This activation can initiate genes that lead to inflammatory responses in the body (Figure 5) [74]. Proteins such as zonula occludens, occludin, and E-cadherin are essential for maintaining the integrity of tight junctions in the intestinal barrier and BBB [75,76,77,78]. Studies in AD have shown alterations in these proteins, suggesting a connection between barrier integrity and neurodegenerative diseases [77,79].

He et al. (2023) investigated the deposition of Aβ in the intestinal epithelium and the measurement of tight junction proteins to assess intestinal permeability [80]. The results showed the accumulation of Aβ in the brain cortex and hippocampus, as well as in intestinal mucosa. There was also a significant decrease in tight junction proteins, including zonula occludens and occludin, compared to controls [80].

In a contradicting study by Qaisar et al. (2023), they investigated plasma zonulin levels in different patient groups, including those with mild AD (*n* = 71), moderate AD (*n* = 66), and a control group (*n* = 74) [68]. Plasma zonulin levels were higher in patients with both mild and moderate AD compared to the control group, with statistical significance.

## 10. Possible Treatment Options Related to Gut Microbiome in Alzheimer’s Disease

Various treatment options for AD have been investigated to be involved in the gut microbiome [81]. For example, prebiotics, which are non-digestible elements, contribute to the growth for a healthy microbiome in the gut by being important components for the production of SCFAs [82]. There is accumulating evidence to employ prebiotics as a means to combat AD through a reduction in Aβ build-up [10,82].

Liu et al. (2021) investigated the role of mannan oligosaccharide using an animal model for AD [83]. The outcomes from immunofluorescence staining revealed a reduction in the accumulation level of Aβ in various regions of the brain including the hippocampus and cortex. In addition, there was an enhancement in the growth for *Lactobacillus* whilst diminishing *Helicobacter* species proliferation. Furthermore, an increase in butyrate formation was observed which is vital to behavioural modification [83].

Another interesting study by Sun et al. (2019) examined the role of fructooligosaccharides, which have an important role in the health of the gut, in an AD animal model [84]. The outcomes showed an enhancement in the cognitive decline using a behavioural evaluation test, including an open-field test (OFT), Morris water maze test (MWM), and object recognition test (ORT); the results were statistically significant. In addition, they measured synaptic plasticity markers including postsynaptic density protein 95 (PSD-95) and synapsin I and found an increase in the expression for these markers. There was an increase in PSD-95, but the result was statistically significant for synapsin I [84].

The probiotics tested were bacterial strains or yeast that help to maintain the health of the gut microbiome [85]. It has been demonstrated that the ingestion of probiotics can enhance the gut microbiome community and has a positive improvement on health [81]. The use of probiotics in AD research has been established and shown to be beneficial to alleviate inflammation [86,87]. Deng et al. (2020) examined over 890 articles related to the use of probiotics as treatment for AD, and yet only 5 studies met their criteria with a total of 287 participants [87]. The outcomes revealed a significant enhancement in the cognitive decline and a significant decrease in malondialdehyde (biomarker for oxidative stress) [87].

Akbari et al. (2016) performed a study on AD patients to evaluate the consumption of probiotics, where 60 patients were tested (30 control = consuming milk, 30 patients = consume combination of probiotics) [86]. The outcomes in the mini-mental state examination (MMSE) displayed a significant improvement in the MMSE score in AD patients compared to the control. Many biomarkers for AD were measured including C-reactive protein, homeostasis model of assessment-estimated insulin resistance, beta cell function, and serum triglycerides, and all were significantly different compared to the control [86].

The use of prebiotics and probiotics is known as synbiotics, and it has been demonstrated to be effective in AD animal models [10]. Deng et al. (2022) examined the use of synbiotics using AD animal models treated with prebiotics and synbiotics compared to transgenic control mice [88]. The MWM test showed that mice treated with prebiotics and synbiotics had shorter time in the maze than controls, but the results were not statistically significant. However, the level of Aβ40 and Aβ42 was assessed using an enzyme-linked immunosorbent assay (ELISA), and the outcomes revealed that Aβ42 was significantly reduced in mice treated with synbiotics compared to control. Nonetheless, the level of Aβ40 has a similar expression in all groups tested [88].

Despite the many advantages of using prebiotics, probiotics, or synbiotics, there is still some confounding drawback for this method. For example, in a previous study, the results from the MWM were not statistically significant, and the levels of Aβ40 were constant, which may indicate that change was due to other confounding factors and was not a real effect of the probiotics or synbiotics [88]. Moreover, most of the studies examined the use of these compounds over a short period of time, and the long-term effect is still unknown. In addition, the response of the probiotics can be highly individualized due to many factors including overall health status or lifestyle [89].

There is escalating evidence that microbial agents might be involved in the pathogenesis of AD including bacteria and viruses [90,91]. Based on the previous theory, the use of antibiotics to help treat AD could be practical. Iizuka et al. (2017) found that rifampicin prevents dementia when administrated in elderly patients with a Clinical Dementia Rating (CDR) less than 1 for one year [92]. However, antibiotic treatment for such a long period of time increases the risk of developing new resistant strains to antibiotics [93]. In addition, the side effect from the long exposure to antibiotics could be deleterious for patients [94].

Faecal microbiota transplantation (FMT) is where the recipient receives a faecal transplant from a donor to change the gut microbiome diversity, and this is usually used as treatment for *Clostridium difficile* [95]. This treatment approach has been investigated using AD animal models and in clinical trials on humans [96,97].

Sun et al. (2019) used APPswe/PS1dE9 transgenic mice of AD to explore the role of FMT [96]. The outcomes showed an enhancement in the cognitive deficit by examining the MWM and ORT, with the mice treated with FMT compared to transgenic mice. In addition, the levels of Aβ40 and Aβ42 were investigated using ELISA, and the results revealed a reduction for both Aβ40 and Aβ42 compared to transgenic mice (control). Moreover, Western blots presented significantly declined tau phosphorylation in the FMT-treated mice compared to transgenic mice (control).

There is an interesting study presented by Hazan (2020) where AD patients were administered with FMT to treat *Clostridium difficile* infection [98]. Two months following FMT, there was a noticeable improvement in the symptoms of AD, including better mental perception. This was followed by a noteworthy enhancement in mood and memory after six months. Moreover, the infection with *Clostridium difficile* was eliminated after 2 months with no symptoms and negative presence in stool sample [98]. Further investigation is required for FMT to determine potential side effects and to implement it on a larger scale beyond individual studies.

## 11. Conclusions

There is mounting evidence from research studies suggesting that the gut microbiome could be involved in the pathogenesis of AD [34,52,60,65,99]. Many mechanisms involved in this process including several studies displaying the gut microbiome community changed at significant levels between AD and controls [13,23,24,25]. In addition, SCFAs, neurotransmitters, LPSs, amyloids, and the inflammation of the gut are known to play vital roles and contribute to the pathogenesis of AD [33,44,55,65,80]. A possible pathway of treatment such as the use of prebiotics, synbiotics, and antibiotics could be promising to reduce cognitive decline [83,87,92]. However, many questions remain to be answered, for example, how differences in the gut microbiome could be beneficial for one host but not to others. In addition, it is known that the gut microbiome changes with host age [27,29]. Why this occurs and what detriment might be experienced if the gut microbiome remained unchanged are unclear. There may not exist a linear relationship (that is to say, there exists uncertainty) between infection and disease development which makes analysis and disease outcome prediction difficult. The specific interaction between pathogens and hosts is shown to lack consistency, and underlying genetic factors and altered pathway responses require dissection through careful experimentation using gene editing and the computational modelling of quantitative omics data. More comprehensive studies are needed to better understand the complexity of the gut microbiome and to investigate the mechanism of action for neurodegenerative diseases. These studies would help to recognize if the gut microbiome could cause the disease, or act as a risk factor, and will add to the infection hypothesis debate. The roles of viruses with other microbiome constituents need to be investigated as they influence its composition and the possible relationship to AD onset.

## Figures and Tables

**Table 1:** Studies investigating the role of the gut microbiome in AD patients compared to controls or other neurodegenerative disorders.

Study	Sample Size	Key Findings
Zhuang et al. (2018) [23]	43 AD patients and 43 age-matched control subjects.	AD patients showed a higher significant presence of *Bacteroidetes* and a lower level of *Actinobacteria* in comparison to the control group.
Liu et al. (2019) [24]	33 AD patients and 32 control individuals.	A significant decrease in *Firmicutes*, a vital phylum known for producing SCFAs, in the AD patients relative to the control group.
Verhaar et al. (2022) [13]	33 AD patients, 21 with MCI, and 116 with SCD.	Significant differences in *Subdoligranulum* and *Phascolarctobacterium* (both *Firmicutes*).
Vogt et al. (2017) [25]	25 AD patients and compared to 25 controls.	A significant decrease in *Firmicutes* and *Bifidobacterium* and an increase in *Bacteroidetes.*

## Data Availability

No new data were created or analysed in this study. Data sharing is not applicable to this article.
